# Supplementary material for: MetaRibo-Seq measures translation in microbiomes
Source: Nat Commun. 2020 Jun 29;11:3268. doi: 10.1038/s41467-020-17081-z (PMC7324362; doi:10.1038/s41467-020-17081-z)
Supplement: Supplementary file 10 — Supplementary Data 7 [file 41467_2020_17081_MOESM10_ESM.zip › File2/Confidence_High_Taxonomy/312886_out.krona.html]

Javascript must be enabled to view this page.

members
magnitude
magnitudeUnassigned
count
unassigned
taxon
rank

312886\_out

2

2
superkingdom
2

phylum
976
2

2
200643
class

order
171549
2

2
171552
family

838
genus
2

1

SRS1041116\_contig\_number\_5028
species
363265

1

SRS049896\_contig\_number\_604
species
1262929
